# The Potential Therapeutic Targets of Anlotinib in Osteosarcoma: Characterization Based on Patient‐Derived Xenografts and Next‐Generation Sequencing

**DOI:** 10.1002/cam4.70416

**Published:** 2024-12-01

**Authors:** Zuoyao Long, Yajie Lu, Minghui Li, Jing Li, Guojing Chen, Fengwei Wang, Qi Wang, Liangbi Xiang, Zhen Wang

**Affiliations:** ^1^ General Hospital of Northern Theater Command Shenyang China; ^2^ Xijing Hospital The Air Force Military Medical University Xi'an China

**Keywords:** combined therapy, osteosarcoma, PDX model, therapeutic targets

## Abstract

**Background:**

The aim of this study was to analyze the potential therapeutic targets of anlotinib using the patient‐derived xenografts (PDX) and evaluate the efficacy of the combination of chemotherapy and anlotinib in osteosarcoma patients before surgery.

**Methods:**

Forty‐three osteosarcoma specimens were used to establish the PDX model in mice, resulting in Twenty‐one PDX successful models. Eventually, six models were randomly selected for the pharmacodynamic experiment. The tumor‐bearing mice were randomly divided into the anlotinib (3 mg/kg) and placebo groups (*n* = 5 each). After treatment, the tumors were harvested and analyzed by immunohistochemistry (IHC) and western blotting.

**Results:**

In PDX model establishment, the tumors from donors with relapse, metastasis or chemoresistance demonstrated higher engraftment capacity. Histology results suggested that anlotinib significantly inhibited the growth of osteosarcoma by inducing mitotic arrest, necrosis and apoptosis, and selective against tumors with high expression of VEGFR2, PDGFRβ and CD31. Based on these results, five osteosarcoma patients who had progressed during NAC were treated with the combination of anlotinib and chemotherapy before surgery, which led to tumor regression in four patients. Next‐generation sequencing showed that most patients with tumor reduction expressed medium or high levels of VEGFR2 and PDGFRβ mRNA. The toxicities were tolerable.

**Conclusions:**

In conclusion, osteosarcoma with high expression of VEGFR2, PDGFRβ and CD31 is more sensitive to anlotinib. However, the potential of synergistic effect of anlotinib and chemotherapy in osteosarcoma patients needs further investigation.

## Graphical abstract

The tumor‐bearing mice of osteosarcoma were randomly divided into the anlotinib (6 mg/kg) and placebo groups (*n* = 5 each). Anlotinib significantly inhibited the growth of osteosarcoma by inducing mitotic arrest, necrosis and apoptosis. It was selective against tumors with high expression of VEGFR2, PDGFRβ and CD31, and the expression levels of these targets decreased after anlotinib treatment. The combination of anlotinib and chemotherapy led to tumor regression in four osteosarcoma patients with chemoresistance. Next‐generation sequencing showed that most patients with tumor reduction expressed medium or high levels of VEGFR2 and PDGFRβ mRNA.

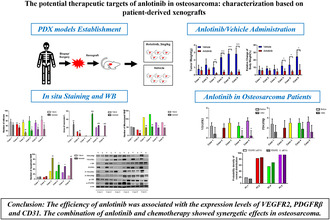

**Abbreviations**
HR: histologic response
IHC: immunohistochemistry
MVD: microvessel density
NAC: neoadjuvant chemotherapy
PDGFR: platelet‐derived growth factor receptor
PDX: patient‐derived xenografts
PFS: progression‐free survival
TKI: tyrosine kinase inhibitor
TV: tumor volume
VEGFR: vascular‐endothelial growth factor

## 1 Introduction

Systematic chemotherapy combined with surgery is the standard treatment regimen for osteosarcoma and can significantly increase the five‐year disease‐free survival from 20% to 60%–70%, along with improving the possibility of limb salvage compared to surgery alone [1, 2]. However, chemo‐resistant patients have poor prognosis even after an active regiment of high‐dose methotrexate, doxorubicin and cisplatin with or without ifosfamide, compared to second‐line chemotherapy [3, 4]. Thus, it is crucial to improve the dismal long‐term survival rate of patients with chemoresistance.

In recent years, drugs targeting angiogenesis‐related signaling pathways have been developed as anti‐tumor agents [5]. Given the critical role of neo‐angiogenesis in tumor growth, invasion and metastasis, drugs specific for angiogenic factors, such as vascular‐endothelial growth factor (VEGFR) and platelet‐derived growth factor receptor (PDGFR), are particularly promising for tumor control [6]. The Italian Sarcoma Group conducted the first phase II clinical trial of the tyrosine kinase inhibitor (TKI) sorafenib in osteosarcoma patients in 2012 [7]. Sorafenib improved the progression‐free survival (PFS) of patients with relapsed and unresectable high‐grade osteosarcoma to 4 months, although a placebo group was not included in this study. Nevertheless, the median PFS of advanced osteosarcoma patients is limited to 3.4–6.2 months, which can be attributed to the high heterogeneity of tumor cells [8, 9]. Therefore, it is essential to explore the potential targets of TKIs in order to select the sensitive patients and improve the long‐term survival.

Anlotinib is an oral TKI that targets multiple kinases such as VEGFR2, fibroblast growth factor receptors (FGFR1‐3), PDGFRβ and c‐Kit and can effectively inhibit tumor growth and vasculature [10]. At present, anlotinib is approved as the second‐ or third‐line treatment for soft tissue sarcoma, renal cell cancer and ovarian cancer [11]. Although anlotinib can significantly inhibit the growth of osteosarcoma cells and xenografts by suppressing VEGFR2 and MET phosphorylation, the prognosis of advanced osteosarcoma patients treated with anlotinib remains controversial [12]. In this study, we established patient‐derived xenografts (PDX) models of osteosarcoma to screen for the potential therapeutic targets of anlotinib and evaluated the synergistic effect of anlotinib and chemotherapy.

## 2 Materials and Methods

### 2.1 Animals

All animals in this study were provided by VitalRiver Biotech (Beijing, China), and the experiments were performed according to standard laboratory animal welfare guidelines (Committee for the Update of the Guide for the Care and Use of Laboratory Animals) of The Fourth Military Medical University, People's Republic of China.

### 2.2 Tumor Tissue Resection and Storage

Tumor tissues consisted of biopsies from primary osteosarcoma or non‐necrotic tumor areas were resected from the patients after neoadjuvant chemotherapy (NAC) or recurrence. The tissues were minced into 3–5 mm pieces, homogenized in RNAStore Reagent (Tiangen Biotech Co. Ltd. Beijing, China), and transferred to liquid nitrogen within 30 min of excision. If the donor was diagnosed with non‐osteosarcoma, the sample was returned or destroyed immediately. Experiments on human samples were approved by the Medical Ethics Committee of the First Affiliated Hospital of the Air Force Medical University and registered in the Chinese Clinical Trial Registry (ChiTR1900026540). Informed consent was obtained from all patients.

### 2.3 PDX Model Establishment

Nu‐Foxn1^Nu^ (Nu‐Nu) immunodeficient mice (6–8 weeks old) were used to establish PDX models. Osteosarcoma tissues frozen in 90% FBS were thawed at 37°C and cut into 2 mm × 2 mm pieces in tissue culture medium under sterile conditions. The tumor pieces were then subcutaneously implanted in the dorsal side of the right forelimbs using a Trocar needle. The tumor‐bearing mice were observed daily for 90 days and the tumor volume (TV) was calculated as length × width^2^/2. The tumors were harvested once the volume exceeded 1000 mm^3^, and implanted into other Nu‐Nu mice. The PDX models were successfully established if the tumors grew after three consecutive passages and used for subsequent analyses.

### 2.4 Anti‐Tumor Activity of Anlotinib in PDX Model of Osteosarcoma

**FIGURE 1:**
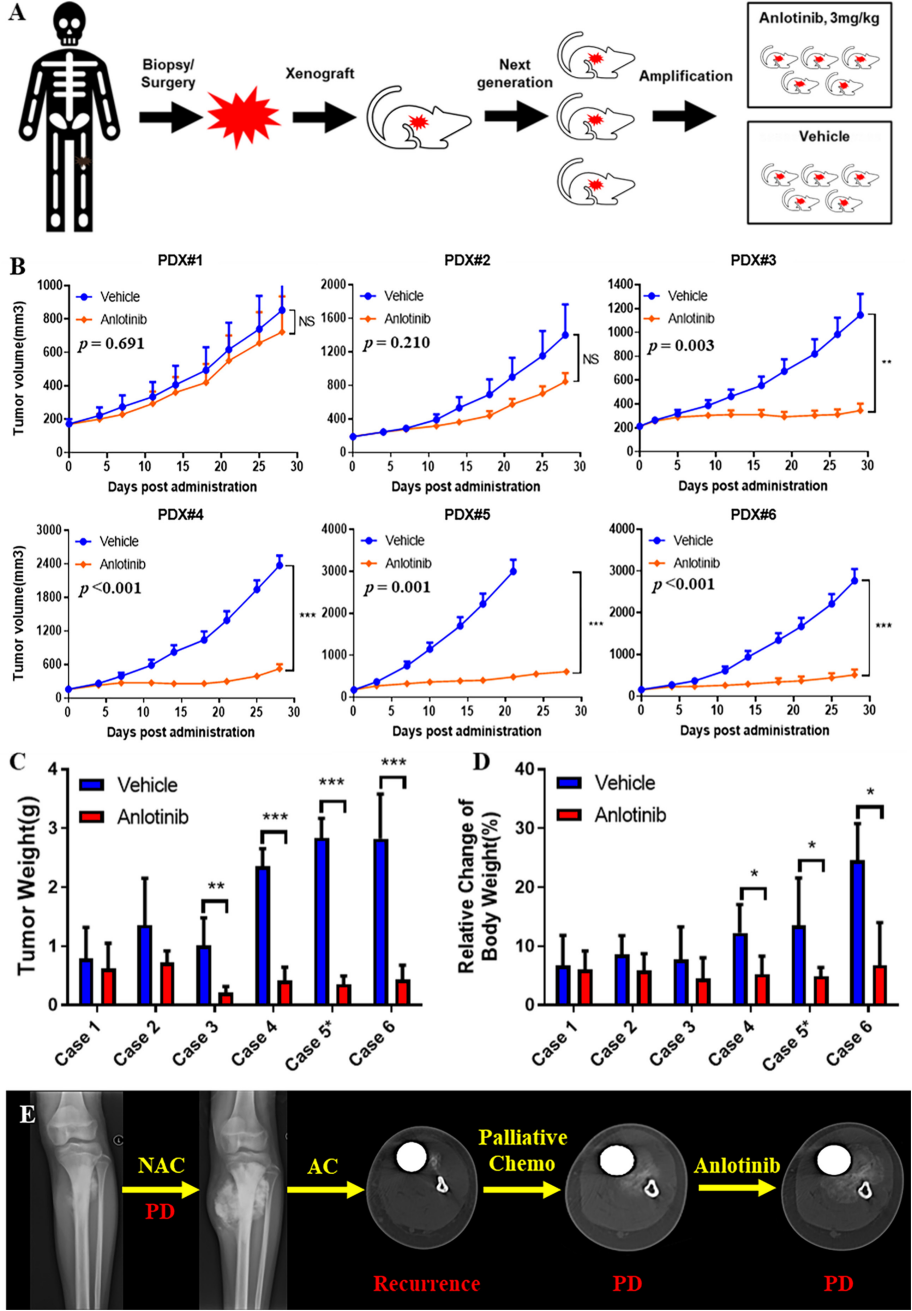
Anlotinib inhibited the growth of osteosarcoma PDX models. (A) Schematic diagram showing osteosarcoma PDX establishment in mice. (B) Tumor volume in the placebo and anlotinib‐treated groups (C) Tumor weight in the indicated groups. (D) Relative change in the body weight of tumor‐bearing mice treated with placebo or anlotinib. (E) The patient, donor of Case 1, was resistant to anlotinib, which was the same as PDX model. *p < 0.05; **p < 0.01; ***p < 0.001.

Six osteosarcoma PDX models were used to evaluate the therapeutic effects of anlotinib. The tumor‐bearing mice were randomly divided into the vehicle and drug groups once the TV was in the range of 150–250 mm^3^ (Figure 1A). Anlotinib (Chia Tai Tianqing Pharmaceutical Group Co.) was dissolved in 0.5% hydroxy propyl methyl cellulose (HPMC) supplemented with 0.2% Tween 80 at a concentration of 0.3 mg/mL. The drug suspension was prepared fresh before each dosage. The mice were administered 10 μL anlotinib suspension or saline intragastrically for a period of 4 weeks. The body weight of the mice was monitored daily and the tumor dimensions were measured every 3 days using a digital caliper. The mice were euthanized in cases of extreme morbidity or when the TV exceeded 3000 mm^3^. Otherwise, tumors were harvested and weighed after 28 days of drug administration. One half of the tumors were frozen at −80°C and the other half was fixed in 4% buffered formaldehyde. The tumor growth inhibition (TGI) rate was calculated as [1−(Δ*T*/Δ*C*)] × 100%, where Δ*T* and Δ*C* refer to the mean TV changes in the drug‐treated and control groups, respectively. TGI > 60% was classified as high response and TGI < 30% as poor response of the PDX models to anlotinib.

### 2.5 In Situ Staining

Formalin‐fixed and paraffin‐embedded tissues were cut into sections. Hematoxylin and eosin (H&E) staining was performed as per the standard protocol, and the histologic response (HR) to treatment and the number of mitotic and apoptotic cells in 10‐high power fields were evaluated. The HR was graded according to the magnitude of necrosis, myxoid degeneration and/or fibrosis as follows: grade 1 (0%–10% of tumor area), grade 2 (11%–50%), grade 3 (51%–90%), and grade 4 (> 90%). For immunohistochemistry (IHC), the sections were probed with antibodies targeting VEGFR2 (ab221679, 1:250), PDGFRβ (ab32570, 1:500), FGFR1 (ab59194, 1:200), and c‐Kit (ab256345, 1:100) (all purchased from Abcam), phosphorylation of VEGFR2 (Tyr1214, 1:1000), PDGFRβ (Tyr740, 1:1000), FGFR1 (Tyr766, 1:1000), and c‐Kit (Tyr721, 1:1000) (purchased from Affinity Biosciences). The number of positively stained cells was counted in at least 5 random fields (400× magnification). The samples were scored on the basis of the percentage of positive cells as: 0—0%; 1—≤ 25%; 2—26%–50%; 3—51%–75%; 4—> 75%. In addition, the staining intensity was scored as follows: 0—no staining; 1—weak staining; 2—moderate staining; 3—intense staining. The final score was calculated by adding the percentage and intensity scores and converted into sum indices as follows: − (0–1), + (2–3), ++ (4–5) and +++ (6–7). Accordingly, the samples were defined as low expression (− or +) or high expression (++ or +++).

Apoptosis was analyzed by terminal deoxynucleotidyl transferase dUTP nick‐end labeling (TUNEL) using a kit (Servivebio Technology CO., LTD., Wuhan, China) according to the manufacturer's instructions. The TUNEL‐positive cells were counted in 5 random fields per slide under 200x magnification using ImageJ Software (Version 1.8.0, National Institutes of Health, USA). The slides were evaluated by two independent pathologists in a blinded manner.

### 2.6 Western Blotting

Western blotting was performed as previously described. The positive bands were visualized and quantified using an electrophoresis image analysis system (Bio‐Rad, Hercules, CA, USA). The antibodies have been listed in the part of IHC staining.

### 2.7 Combination Therapy in Osteosarcoma Patients

Patients were recruited to receive chemotherapy combined with anlotinib as follows: (1) aged 12–30 years; (2) histologically diagnosed as osteosarcoma; (3) progressive disease (PD) after 1–2 cycles of NAC; (4) the Eastern Cooperative Oncology Group performance status score was 0–2, and the expected survival was more than 3 months. The regimen of chemotherapy consists of an infusion of 15 g/m^2^ of ifosfamide, followed by an infusion of 60–75 mg/m^2^ of doxorubicin and 100–120 mg/m^2^ of cisplatin. Anlotinib (12 mg/d) was received on the first day of chemotherapy as part of the schedule of 2 weeks on/1 week off. PD was defined as a tumor increased at least 20% in longest diameter or the development of new lesions. Surgery was recommended after receiving four cycles of chemotherapy totally, or in case the patient did not respond to the treatment. The schedule of anlotinib administration was 12 mg once daily on a 2‐week on and 1‐week off. All patients were evaluated according to Response Evaluation Criteria in Solid Tumors (RECIST) version 1.1 based on imaging results. This study was approved by the Medical Ethics Committee of the First Affiliated Hospital of the Air Force Medical University (YS20181014‐C‐1).

All drug‐related adverse events were carefully recorded since the beginning of combined therapy. The severity was assessed according to the CTCAE v5.0. Dose adjustment was carried out according to the specifications when grade ≥ 3 AEs occurred.

### 2.8 Statistical Analysis

Statistical analysis was performed with SPSS v 26.0 software (IBM SPSS Inc., Armonk, NY, USA). Categorical variables were compared by the chi‐square test or Fisher's exact test, and continuous variables were compared using the unpaired two‐tailed *t* test or one‐way ANOVA. The pathological features were analyzed by the Mann–Whitney test and the Kruskal–Wallis test. *p* < 0.05 was considered statistically significant.

## 3 Results

### 3.1 Establishment of PDX Models of Osteosarcoma

Tumor specimens were collected from 43 osteosarcoma patients, and the baseline characteristics of the patients are summarized in Table 1. Twenty‐one osteosarcoma PDXs were generated successfully, representing an engraftment rate of 48.84%. Among these established PDXs, 6 models (28.57%) were derived from locally relapsed tissue compared to the failed group (*n* = 1/22, 4.55%), which demonstrated a higher success rate of graft with relapsed tissue (*p* = 0.046). In addition, distal metastasis was also positively correlated with successful modeling (47.62% vs. 0%, *p* < 0.001). We also observed higher engraftment capacity of tumors that had responded poorly to chemotherapy. Only 1 of 8 specimens from patients with PD failed to grow in mice (*p* = 0.013). Therefore, we hypothesized that tumors with higher invasiveness, including relapse, distal metastasis or chemotherapy resistance, would demonstrate greater engraftment capacity.

**TABLE 1:** Patient demographic and baseline characteristics.

Characteristic	All	Succeed modeling	Failed modeling	OR (95%CI)	*p* *
No.	43	21	22		
Gender, male (%)	53.49	57.14	50.00	1.14 (0.65–2.00)	0.639
Age, years (%)					
Median age	15	15 (5–43)	15 (6–45)		
Age < 15	48.84	52.38	45.45	1.15 (0.62–2.13)	0.650
Location (%)					
Upper limb	9.30	9.52	9.09	1	1.00
Lower limb	83.72	80.96	86.36	0.99 (0.80–1.22)	0.916
Other	6.98	9.52	4.55	1.50 (0.23–9.80)	1.00
Tumor size, > 10 cm (%)	51.16	47.62	54.55	0.87 (0.48–1.57)	0.650
Relapsed tissue, yes (%)	16.28	28.57	4.55	6.29 (1.20–47.90)	0.046
Metastasis, yes (%)	23.26	47.62	0	—	< 0.001
Response to chemo (%)					
PR&SD	55.82	38.10	72.73	1	1.00
PD	18.60	33.33	4.55	7.93 (1.10–57.28)	0.013
No chemo	25.58	28.57	22.73	1.8 (0.68–4.77)	0.234
Chemo before tissue collection, yes (%)	44.19	42.86	45.45	0.94 (0.48–1.85)	0.864

* Chi square test (Fisher exact test).

### 3.2 Sensitivity of the PDX Models Toward Anlotinib Treatment

Anlotinib demonstrated various responses in PDX models. We successfully established 6 PDX models, including 2 from relapsed tumor specimens (PDX#4 & 5), 1 from primary tumor with distal metastasis (PDX#2) and 3 from progressed tumors after NAC (PDX#1, 3 & 6). The histological features of the PDX tissues were comparable to those of the corresponding patient tumor tissues. Anlotinib achieved poor response in PDX#1, medium response in PDX#2, and good response in PDX#3–6. Anlotinib significantly inhibited tumor growth compared to that in the vehicle group in terms of both volume (Figure 1B) and weight (Figure 1C). During the treatment, the mice had a stable body weight within ethically acceptable limits (Figure 1D), and no adverse events were observed.

### 3.3 Validation of Potential Therapeutic Targets in PDX Models

**FIGURE 2:**
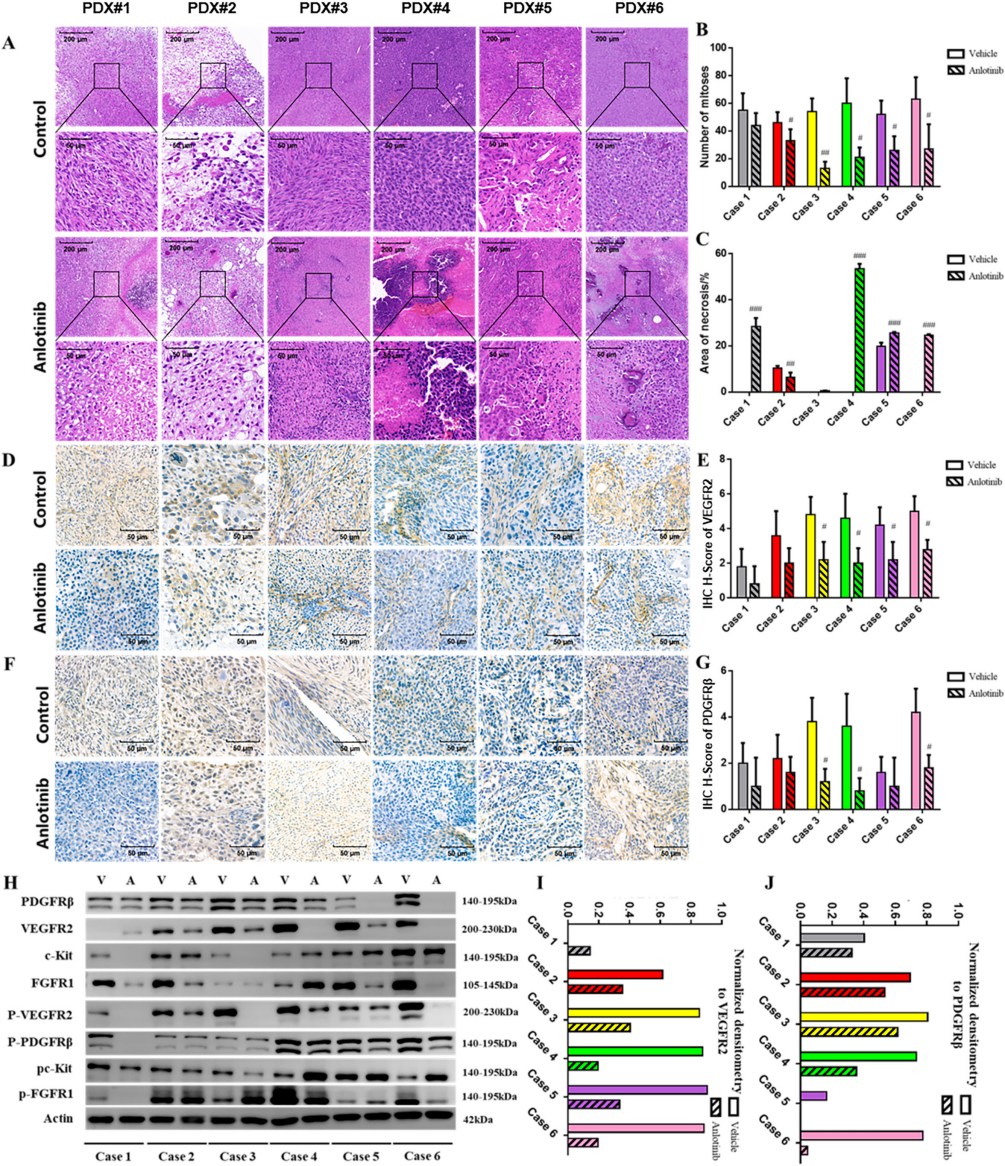
Pathological response of the osteosarcoma PDXs to anlotinib. (A) Representative images of HE‐stained tissues of PDX models. (B) Number of mitoses in the placebo and anlotinib‐treated groups. (C) Tumor necrosis in the different PDX models. (D, E) VEGFR2 expression in the indicated groups (score 0–6). (F, G) PDGFRβ expression in the indicated groups (score 0–5). (H) Immunoblot showing expression levels of angiogenesis‐related factors before and after anlotinib treatment. (I, J) Quantification of VEGFR2 and PDGFRβ levels. #*p* < 0.05; ##*p* < 0.01; ###*p* < 0.001.

**FIGURE 3:**
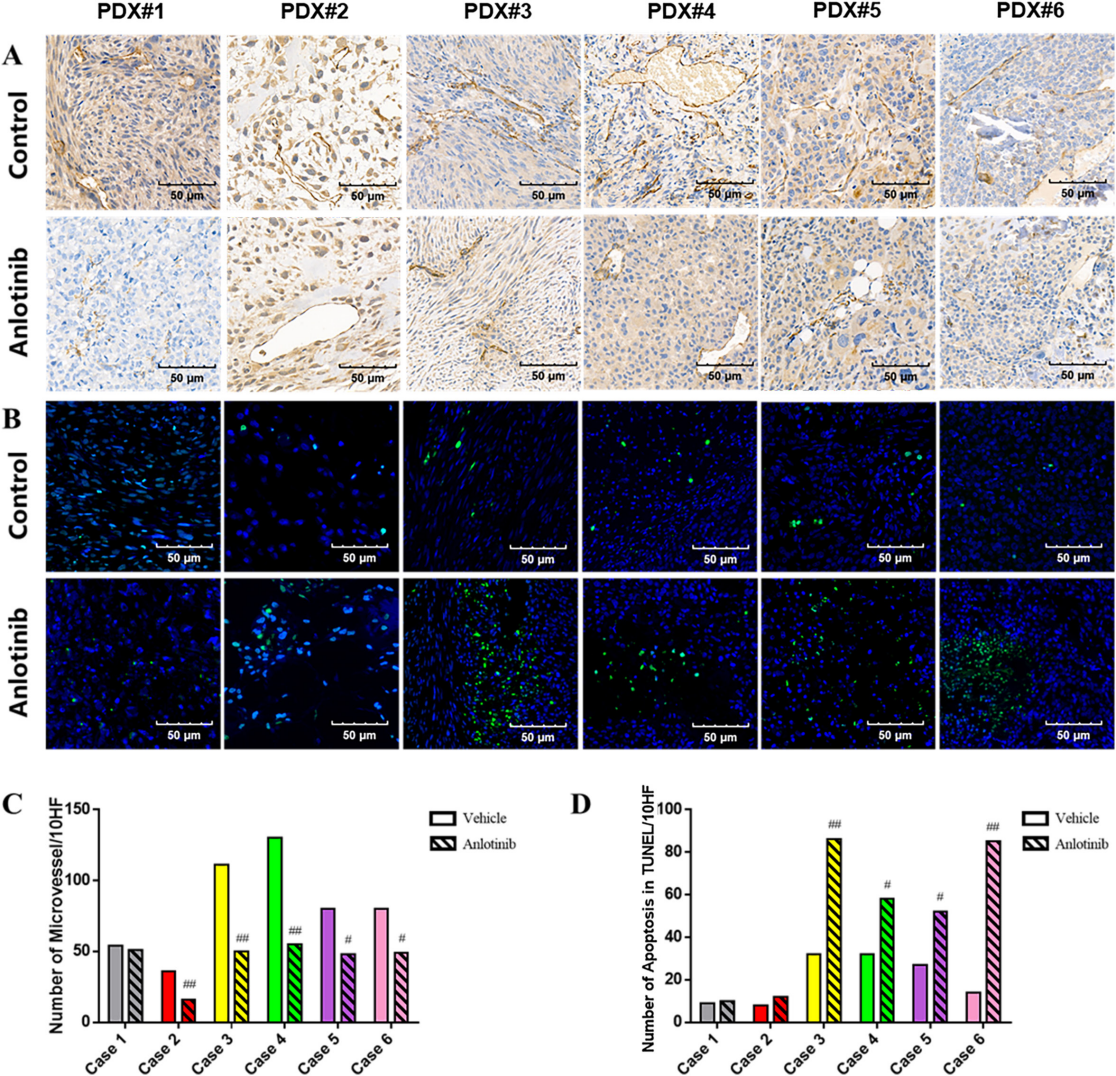
High microvessel density sensitized the osteosarcoma PDX models to anlotinib. (A, C) Representative images of PDX tissues showing CD31+ microvessels and histological characteristics before and after anlotinib treatment. (B, D) Representative images showing TUNEL+ (green fluorescence) apoptotic cells in the PDX tissues. #*p* < 0.05; ##*p* < 0.01; ###*p* < 0.001.

Histological staining showed that most vehicle‐treated tumors had minimal (grade 1) HR, whereas anlotinib significantly decreased the number of mitotic tumor cells (*p* < 0.05) and increased tumor necrosis, including a grade 3 HR in PDX#4 (*p* < 0.0001, Figure 2A–C). Notably, PDX models that were sensitive to anlotinib showed a more substantial reduction in mitosis (Figure 2B). Furthermore, anlotinib selectively inhibited the growth of PDX models with high VEGFR2 or PDGFRβ expression (Figure 2D–J), and even downregulated both targets, especially in the responsive xenografts. In addition, the expression of phosphorylated VEGFR2 and PDGFRβ were also downregulated in the anlotinib‐treated tumors, which was consistent with previous reports. We also evaluated the microvessel density (MVD) based on CD31 expression and found that tumors with high MVD were more sensitive to anlotinib. The MVD also decreased significantly after anlotinib treatment and is therefore a potential predictor of anlotinib response (Figure 3A,C). Finally, the percentage of TUNEL‐positive apoptotic cells was also higher in the PDX models with good response (PDX#3–6) and decreased significantly after anlotinib treatment. This finding suggested that anlotinib had multifaceted efficiency in osteosarcoma PDX models, involving inhibition of tumor proliferation, induction of necrosis, and modulation of angiogenesis and apoptosis. In addition, the expression of VEGFR2, PDGFRβ, and MVD could potentially serve as predictive biomarkers for determining the response of anlotinib.

### 3.4 Combination Therapy of Anlotinib and NAC in Chemo‐Resistant Osteosarcoma Patients

Between January 1, 2020 and December 31, 2020, 5 patients (4 males and 1 female) were recruited to receive the combination of anlotinib and NAC. The characteristics of the patients are summarized in Table 2. Four patients had tumor regression (8.7% to 69.4%) after the combination treatment, of which two achieved partial response (PR). The remaining patient showed 19.3% tumor progression after one‐cycle of the combination therapy. All patients had undergone complete resection of the primary tumor and received the same regimen of anlotinib and adjuvant therapy except for the progressed patient.

**TABLE 2:** Clinical characteristics of osteosarcoma patients with anlotinib + chemotherapy.

No.	Gender	Age	Site	Diagnosed		Chemotherapy alone			Anlotinib + chemotherapy			Surgery
				Size^a^ (cm)	Metastasis	Cycles	Size (cm, %)	Metastasis	Cycles	Size (cm, %)	Metastasis	
Pt.1	M	14	Left femur	2.8	NA	1	8.5 (203.6%)	NA	3	2.6 (−69.4%)	NA	R0
Pt.2	F	12	Left tibia	0	NA	2	2.5	NA	2	1.8 (−28.0%)	NA	R0
Pt.3	M	19	Right fibula	0.8	NA	2	3.1 (287.5%)	NA	1	3.7 (19.3%)	NA	R0^b^
Pt.4	M	27	Right femur	12.3	NA	2	14.9 (21.1%)	NA	2	13.6 (−8.7%)	NA	R0
Pt.5	M	16	Right femur	5.3	Yes	2	12.1 (128.3%)	PD	2	9.8 (−19.0%)	PR	R0

^a^ Tumor size was defined as the longest diameter of soft tissue lesion.

^b^ Patient No. 3 refused to receive anlotinib + chemotherapy and performed surgery in another hospital.

**FIGURE 4:**
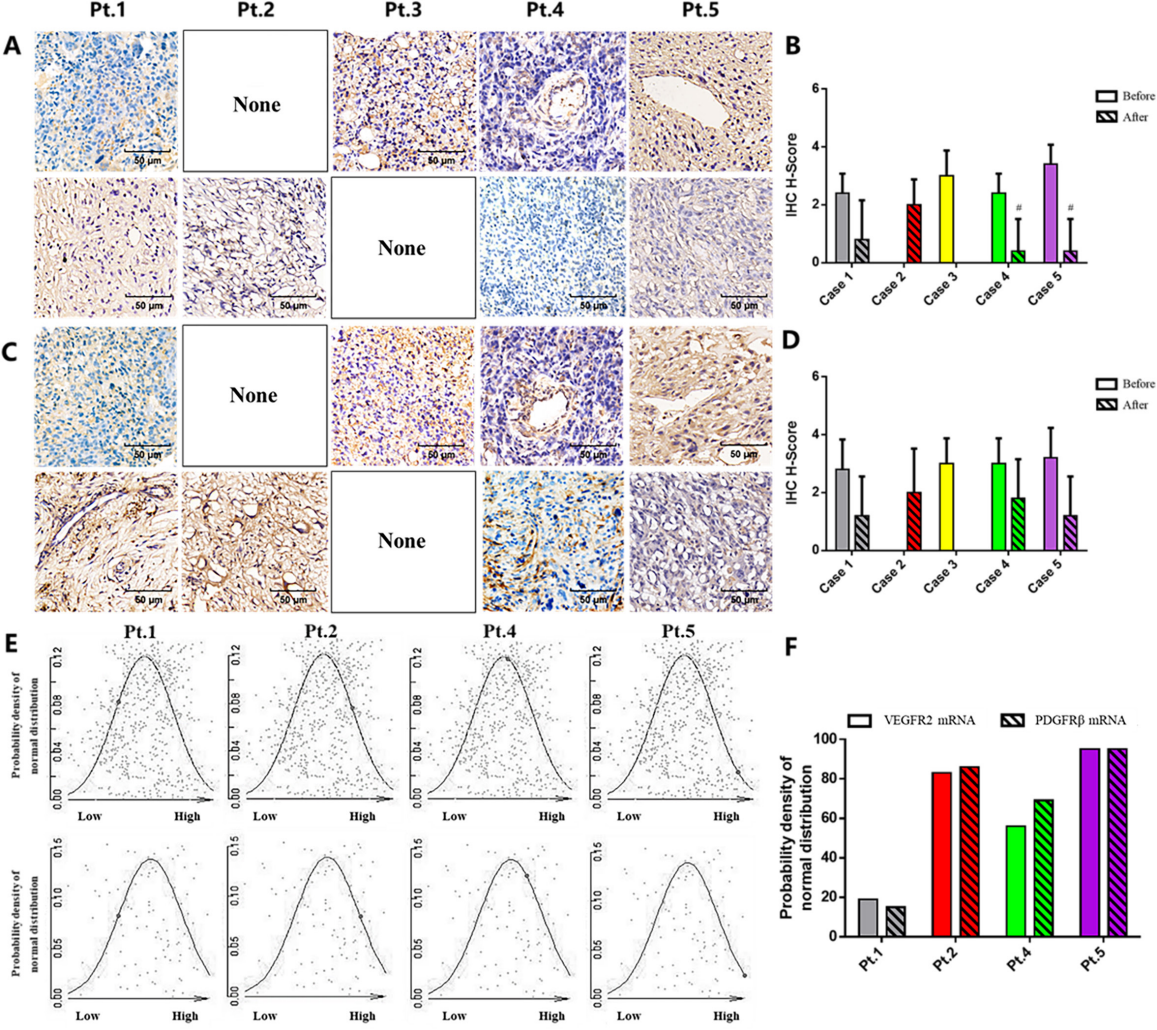
The expression of VEGFR2 and PDGFRβ in osteosarcoma patients. (A, B) Representative images of osteosarcoma tissues showing in situ VEGFR expression. (C, D) Representative images of osteosarcoma tissues showing in situ PDGFRβ expression. (E, F) VEGFR2 and PDGFRβ mRNA levels in the different patients. #*p* < 0.05.

Retrospectively, we collected the tumor tissue of these patients. As observed in the PDX tissues, the in situ expression of both VEGFR2 and PDGFRβ was reduced after anlotinib treatment (Figure 4A–D). Furthermore, next‐generation sequencing revealed that most patients with tumor reduction expressed medium or high levels of VEGFR2 and PDGFRβ mRNA, although there was no significant difference in their expression in biopsy tissues (Figure 4E,F). These results provided insights into the efficiency of anlotinib in combination with NAC for treating osteosarcoma with chemotherapy resistance. Further investigation was needed to explore the synergistic effect of anlotinib and chemotherapy.

Although the combination therapy caused superimposed toxicities, most of the adverse events were grade 1 or 2, and were tolerable. The most common grade 1–2 adverse events were fatigue, anorexia, and loss of weight. Myelosuppression was the most severe grade 3 adverse event that occurred in two patients but was alleviated by treating the patients with human granulocyte colony stimulating factor. None of the patients had to undergo dose adjustment.

## 4 Discussion

Anlotinib is a multiple TKI that targets VEGFR2 and PDGFRβ and therefore exhibits broad‐spectrum anti‐tumor activity [13]. This study was designed to investigate the potential therapeutic targets of anlotinib in osteosarcoma using PDX models. Our findings indicated that anlotinib selectively inhibited osteosarcoma with high expression of VEGFR2, PDGFRβ or CD31.

Although PDX models retain the characteristics and heterogeneity of the donor tumors, there are two factors that restrict their wider application in preclinical studies [14]. First, establishment of a PDX model takes 6 months–2 years, which is unpractical in the case of patients in the advanced stages of cancer [15]. Second, the success rate varies from 10% to 90%, depending on the histological type and implantation site. The overall engraftment efficiency of osteosarcoma tissues is reportedly 20% to 40% [16]. In addition, the engraftment rate of PDXs is significantly reduced after chemotherapy, and a strong negative correlation has been observed between the 3‐year survival probability of the donors and PDX engraftment [17]. In the present study, we successfully established 21 PDX models in Nu‐Nu immunodeficient mice with a success rate of 48.84%, which was higher than that reported by previous studies. Furthermore, all PDXs were modeled within 3 months. In addition, the tumor specimens obtained from patients with relapse, drug resistance or metastasis showed greater engraftment capacity. Nevertheless, the procedure is still time‐consuming and costly, which needs further improvement.

Given the crucial role of angiogenesis in tumor progression and metastasis, various anti‐angiogenic drugs have been developed in recent years to treat patients recalcitrant to conventional chemotherapy [5]. These drugs mainly target the VEGF/VEGFR pathway, either by inhibiting kinase activity or blocking ligand‐receptor binding [18]. Bevacizumab is a broad‐spectrum humanized anti‐VEGF antibody that can induce proliferation of endothelial cells in vitro but has not been effective in osteosarcoma patients [19]. On the other hand, TKIs such as sorafenib and regorafenib can inhibit osteosarcoma progression in vivo [20, 21]. In this study, anlotinib inhibited the growth of PDX models by varying degrees and led to significant shrinkage in the tumor mass in four models (Figure 1B,C). Furthermore, the sensitivity of the PDXs to anlotinib correlated positively with tumor necrosis, apoptosis and mitosis inhibition (Figures 2A–C and 3B,D). The safety of anlotinib was acceptable considering that no severe weight loss or drug‐related death occurred during the treatment.

The substantial primary resistance to TKIs has promoted the identification of novel therapeutic targets associated with angiogenic factors or cell cycle. Chen et al. showed that volitinib was highly effective against gastric PDX models overexpressing MET and pMET, whereas EGFR or HER2 overexpression was associated with greater sensitivity to afatinib [22]. Furthermore, the highly‐selective VEGFR2 inhibitor apatinib is effective against tumors with high MVD, and its efficacy depends on VEGFR‐2 expression [23]. In our study, anlotinib inhibited the growth of PDX models with high expression of VEGFR2 or PDGFRβ, and the expression of both receptors decreased after anlotinib treatment. Moreover, the MVD was a predictive marker of the response to anlotinib. Due to the limited number of the PDX models, however, the cut‐off value of CD31 expression needs further confirmation.

Anti‐angiogenesis therapy promotes vascular normalization and alleviates the anoxic environment in tumors, which enhances the delivery and efficacy of concurrently administered drugs [24]. Thus, the combination of anti‐angiogenic TKI combined with chemotherapy is a promising anti‐cancer strategy. For instance, chemotherapy combined with an EGFR inhibitor has been widely used to treat non‐small cell lung cancer; likewise, regorafenib has shown favorable effects in the combination therapy of metastatic colorectal cancer [25, 26]. We had previously reported that anlotinib in combination with adriamycin and ifosfamide improved tumor regression and the rate of limb‐salvage surgery in patients with unresectable soft tissue sarcoma [27]. Furthermore, including anlotinib in the gemcitabine/docetaxel regimen prolonged the mPFS of advanced osteosarcoma patients from 5 months to 9 months (*p* = 0.048) [28]. However, the efficacy of preoperative combination therapy in osteosarcoma has been rarely reported. Therefore, we exploratively recruited five patients who had progressed during NAC and treated them with anlotinib plus first‐line chemotherapy. Two patients achieved PR, two had stable disease (SD) with tumor shrinkage, and one had SD with tumor growth. All patients underwent salvage surgery and combination maintenance. Next‐generation sequencing showed that patients with medium or high expression of VEGFR2 and PDGFRβ mRNA responded to the combination therapy, which may be used as predictors of treatment response.

One of the limitations in our study was the lack of secondary‐resistant PDX models. The expression levels of the anlotinib targets were associated with tumor response and decreased after the treatment, which may promote secondary resistance. However, the TKI‐induced change in the levels of targets is still controversial. In fact, studies using cellular models have shown that anlotinib does not inhibit the expression of VEGFR2 [10, 13], which contradicts our findings and those reported by Li et al. in an in vivo model [28, 29]. Furthermore, the number of patients was limited since the evidence presented in this study was insufficient to select patients for combined therapy. Thus, a prospective trial with a large cohort should be conducted to validate our findings. Finally, it remains to be confirmed whether adding anlotinib to NAC could improve the long‐term survival of osteosarcoma patients with chemotherapy resistance.

## 5 Conclusion

In conclusion, anlotinib inhibited the growth of osteosarcoma PDX models by varying degrees depending on the expression levels of VEGFR2, PDGFRβ and CD31. The next focus would be to screen for patients sensitive to anlotinib and evaluate the correlation between target expression and drug resistance. However, the potential synergistic effect of anlotinib and chemotherapy in osteosarcoma patients needs further investigation.

## Author Contributions

**Zuoyao Long:** data curation (lead), formal analysis (lead), investigation (lead), software (lead), validation (lead), visualization (lead), writing – original draft (lead), writing – review and editing (lead). **Yajie Lu:** data curation (equal), formal analysis (equal), investigation (equal), validation (equal), writing – review and editing (equal). **Minghui Li:** data curation (equal), formal analysis (equal), investigation (equal), writing – review and editing (equal). **Jing Li:** data curation (equal), resources (equal), validation (equal). **Guojing Chen:** resources (equal), validation (equal). **Fengwei Wang:** resources (equal), validation (equal). **Qi Wang:** project administration (equal), supervision (equal). **Liangbi Xiang:** conceptualization (equal), project administration (equal), supervision (equal). **Zhen Wang:** conceptualization (equal), methodology (equal), supervision (equal).

## Ethics Statement

This study was approved by the Medical Science Committee of the First Affiliated Hospital of Air Force Medical University and registered in the Chinese Clinical Trial Registry (ChiTR1900026540).

## Consent

Written informed consent was provided by all participants prior to inclusion in the study.

## Conflicts of Interest

The authors declare no conflicts of interest.

## Data Availability

Data archiving is not mandated but data will be made available on reasonable request.
